# Association Between Cardiac Autonomic Function and Peripheral Nerve Conduction Abnormalities in Type 2 Diabetes Mellitus: A Cross-Sectional Study

**DOI:** 10.7759/cureus.107133

**Published:** 2026-04-15

**Authors:** Anwar H Siddiqui, Md S Alam, Ahmad Faraz, Nazia Tauheed, Hamid Ashraf, SAA Rizvi

**Affiliations:** 1 Department of Physiology, Jawaharlal Nehru Medical College and Hospital, Aligarh Muslim University, Aligarh, IND; 2 Department of Anesthesiology, Jawaharlal Nehru Medical College and Hospital, Aligarh Muslim University, Aligarh, IND; 3 Department of Endocrinology, Diabetes, and Metabolism, Jawaharlal Nehru Medical College and Hospital, Aligarh Muslim University, Aligarh, IND

**Keywords:** autonomic dysfunction, cardiac autonomic neuropathy, glycemic control, heart rate variability, nerve conduction study, peripheral neuropathy, type 2 diabetes

## Abstract

Background: Cardiac autonomic neuropathy and diabetic peripheral neuropathy often develop early in type 2 diabetes mellitus (T2DM) and may remain clinically silent. Heart rate variability (HRV) and nerve conduction studies (NCS) are noninvasive tools that assess autonomic and peripheral nerve function, respectively. Their relationship in early-stage T2DM is not well defined.

Methods: This cross-sectional study included 100 patients with T2DM of less than five years’ duration (age 30-50 years) and 100 age- and sex-matched healthy controls. Five-minute resting HRV recordings were analyzed for time- and frequency-domain parameters. Motor and sensory NCS were performed for the median, posterior tibial, and sural nerves. Group comparisons, correlation analysis, and multivariable regression were conducted to evaluate associations and predictors of HRV impairment.

Results: T2DM patients demonstrated significantly reduced parasympathetic HRV indices, including root mean square of successive differences and high-frequency (HF) power (p ≤ 0.001), along with reduced sensory and motor nerve amplitudes, most prominently sural SNAP amplitude (p < 0.001). HRV parameters showed positive correlations with NCS measures, with the strongest association observed between HF power and sural SNAP amplitude (r = 0.62). Multivariable regression identified higher glycated hemoglobin and longer diabetes duration as independent predictors of reduced HRV, while age, sex, and body mass index were not significant.

Conclusions: Early-stage T2DM is associated with parallel autonomic and peripheral nerve dysfunction. HRV correlates with nerve conduction abnormalities, suggesting shared pathophysiology. Poor glycemic control and longer disease duration independently predict autonomic impairment, highlighting the importance of early metabolic control and combined neurophysiological assessment for detecting subclinical neuropathy.

## Introduction

Diabetes mellitus (DM) is a major global public health concern and a leading cause of morbidity and mortality worldwide. It significantly impairs quality of life and was estimated to contribute to approximately six million deaths in 2021. India, often referred to as the “diabetic capital of the world,” has witnessed a rapid increase in disease burden, with the number of affected individuals rising from 40.9 million in 2007 to 77 million in 2019, and projections suggesting further escalation in the coming decades [1]. Type 2 diabetes mellitus (T2DM), which accounts for nearly 90% of all diabetes cases globally, is characterized by insulin resistance and progressive β-cell dysfunction, resulting in chronic hyperglycemia and long-term microvascular and macrovascular complications [2].

Among these complications, cardiac autonomic neuropathy (CAN) and diabetic peripheral neuropathy (DPN) are particularly important due to their association with significant functional impairment and adverse outcomes. CAN results from damage to autonomic nerve fibers regulating heart rate and vascular tone, leading to abnormalities in cardiovascular reflexes and increasing the risk of arrhythmias, silent ischemia, and sudden cardiac death [3]. Importantly, CAN often remains subclinical until advanced stages, making early recognition challenging in routine practice [4]. Although cardiovascular autonomic reflex tests are considered the gold standard for diagnosis, their time-consuming nature and requirement for patient cooperation may limit widespread clinical application [5].

Heart rate variability (HRV), which reflects beat-to-beat fluctuations in cardiac rhythm, has emerged as a practical and noninvasive method for assessing autonomic function. Reduced HRV has been associated with impaired parasympathetic activity and is recognized as an indicator of autonomic dysfunction as well as an independent predictor of cardiovascular morbidity and mortality in individuals with diabetes [6]. In parallel, DPN represents the most common microvascular complication of DM, affecting nearly half of patients over the course of the disease. It typically manifests as a length-dependent sensory-motor neuropathy, resulting in sensory loss, motor weakness, and increased risk of foot complications [7]. Nerve conduction studies (NCS) provide an objective and sensitive evaluation of peripheral nerve integrity through measurements of latency, amplitude, and conduction velocity, and remain an established tool for detecting subclinical neuropathic changes [8].

While HRV and NCS are routinely used to evaluate autonomic and peripheral nerve function separately, the relationship between these modalities in early-stage T2DM has not been extensively investigated. Understanding whether alterations in autonomic function parallel peripheral nerve abnormalities may provide additional insight into shared pathophysiological mechanisms. Furthermore, glycated hemoglobin (HbA1c), a marker of long-term glycemic control, has been linked to subclinical cardiovascular and neural dysfunction, although its independent association with autonomic and peripheral nerve changes requires further clarification [9].

Therefore, the present study aimed to evaluate HRV and NCS parameters in patients with short-duration T2DM, examine the association between autonomic and peripheral nerve function, and identify clinical predictors, including HbA1c and disease duration, that may contribute to early neurophysiological impairment.

## Materials and methods

Study design and setting

This cross-sectional study was conducted in the Department of Physiology and the Center for Diabetes and Endocrinology of a tertiary care teaching hospital. The study evaluated the association between cardiac autonomic function assessed by HRV and peripheral nerve function assessed by NCS in patients with T2DM. Institutional Ethics Committee approval was obtained prior to commencement of the study (approval no: IECJNMC/1253, dated December 25, 2023). The study was conducted between January 2024 and February 2025.

Study population

A total of 100 patients with confirmed T2DM of less than five years’ duration were recruited from the outpatient department. To focus on early-stage disease and minimize age-related autonomic changes, only patients aged 30-50 years were included. One hundred age- and sex-matched healthy individuals without diabetes were enrolled as controls. Controls were screened using fasting plasma glucose and HbA1c to exclude undiagnosed diabetes. Institutional ethics committee approval was obtained, and written informed consent was secured from all participants.

Inclusion and exclusion criteria

Inclusion criteria comprised diagnosed T2DM of less than five years’ duration, age 30-50 years, stable medication for at least three months, and willingness to undergo HRV and NCS testing.

Exclusion criteria included type 1 diabetes, diabetes duration of at least five years, peripheral arterial disease, systemic conditions known to cause neuropathy (vitamin B12 deficiency, malnutrition, alcoholism, and renal failure), exposure to neurotoxic drugs or metals, active infection, pregnancy, or any other neurological disorder.

Diagnostic criteria for T2DM

T2DM diagnosis was based on the revised American Diabetes Association criteria [10], including fasting plasma glucose ≥126 mg/dL, two-hour plasma glucose ≥200 mg/dL during oral glucose tolerance testing, HbA1c ≥6.5%, or random plasma glucose ≥200 mg/dL with symptoms.

Sample size

The sample size was calculated to detect a moderate correlation (r = 0.3) between HRV and NCS parameters with 80% power and α = 0.05 [11]. The minimum required sample was 85 subjects per group. To account for potential attrition, 100 participants were included in each group.

Clinical and biochemical assessment

Participants attended the laboratory after an overnight fast of 10-12 hours. Fasting plasma glucose, postprandial glucose, and HbA1c were measured. Plasma glucose was estimated using the glucose oxidase-peroxidase method, and HbA1c was measured using high-performance liquid chromatography calibrated to National Glycohemoglobin Standardization Program standards.

HRV assessment

HRV was recorded using a PowerLab data acquisition system (AD Instruments, Australia) with a sampling rate of 1,000 Hz. Participants were instructed to abstain from caffeine, alcohol, smoking, and vigorous physical activity for at least 24 hours prior to testing. Recordings were performed in a quiet, temperature-controlled room (22°C-24°C) with subjects in the supine position after a 10-minute rest period.

A continuous five-minute ECG recording was obtained using a standard lead II configuration. Time-domain parameters, including standard deviation of NN intervals (SDNN), root mean square of successive differences (RMSSD), and pNN50, were calculated. Frequency-domain analysis was performed using fast Fourier transform to obtain low-frequency (LF: 0.04-0.15 Hz) and high-frequency (HF: 0.15-0.40 Hz) components expressed in meter per square second. The LF/HF ratio was calculated to assess sympathovagal balance [12].

Nerve conduction studies

NCS were performed using a NeuroPerfect EMG/NCV system (Medicaid Systems, Chandigarh, India). Motor nerve amplitudes were measured as compound muscle action potential (CMAP) amplitudes recorded from the abductor pollicis brevis for the median nerve and abductor hallucis for the posterior tibial nerve. Sensory nerve amplitudes were recorded as sensory nerve action potential (SNAP) amplitudes using antidromic stimulation for the median nerve and standard surface recording for the sural nerve.

Amplitudes were measured peak-to-peak in millivolts for motor nerves and microvolts for sensory nerves. Standard electrode placement and stimulation techniques were followed according to established electrophysiological guidelines [13].

Statistical analysis

Data were analyzed using Statistical Package for the Social Sciences version 25 (IBM Corp., Armonk, NY). Continuous variables were expressed as mean ± standard deviation or median (interquartile range) based on distribution assessed using the Shapiro-Wilk test. Group comparisons were performed using independent t-tests or Mann-Whitney U tests as appropriate.

Correlations between HRV and NCS parameters were assessed using Pearson’s or Spearman’s correlation coefficients. Multiple linear regression analysis was used to identify independent predictors of HRV indices, including HbA1c, diabetes duration, age, body mass index, and sex. Effect sizes and model R² values were reported. A p value of <0.05 was considered statistically significant.

## Results

The demographic and clinical characteristics of 100 patients with T2DM and 100 age- and sex-matched healthy controls are summarized in Table 1.

**Table 1:** Demographic and clinical characteristics of study participants Values are expressed as mean ± SD unless otherwise indicated. An independent samples t-test was used for continuous variables and a chi-square test for categorical variables T2DM: type 2 diabetes mellitus; BMI: body mass index; SD: standard deviation; HbA1c: glycated hemoglobin

Variable	T2DM patients (n = 100)	Healthy controls (n = 100)	p value
Age (years)	46.4 ± 4.2	44.8 ± 5.4	0.66
Sex (male/female)	54/46	58/42	0.82
Duration of diabetes (years)	3.9 ± 1.2	-	-
BMI (kg/m²)	27.6 ± 2.9	25.7 ± 2.5	0.02
HbA1c (%)	7.8 ± 1.4	5.2 ± 0.4	<0.001
Fasting glucose (mg/dL)	143.7 ± 28.2	95.3 ± 7.8	<0.001

The two groups were comparable with respect to age and sex distribution. As expected, T2DM patients had significantly higher fasting glucose, HbA1c, and body mass index than controls (p < 0.05). The mean duration of diabetes in the patient group was less than five years, reflecting early-stage disease. HRV parameters are presented in Table 2.

**Table 2:** HRV parameters in type 2 diabetes mellitus patients and healthy controls Values are expressed as mean ± SD. p values were calculated using independent samples t-tests. Cohen’s d represents the effect size HRV: heart rate variability; T2DM: type 2 diabetes mellitus; SDNN: standard deviation of NN intervals; RMSSD: root mean square of successive differences; LF: low frequency; HF: high frequency; SD: standard deviation

HRV parameter	T2DM patients (n = 100)	Healthy controls (n = 100)	p value	Cohen’s d
Time-domain parameters				
SDNN (ms)	37.5 ± 10.2	47.3 ± 12.5	0.136	0.43
RMSSD (ms)	21.5 ± 8.2	35.7 ± 9.1	0.001	1.24
pNN50 (%)	7.2 ± 3.1	12.6 ± 3.8	0.032	0.88
Frequency-domain parameters				
LF power (ms²)	394 ± 120	476 ± 143	0.001	0.74
HF power (ms²)	154 ± 56	283 ± 78	0.001	1.67
LF/HF ratio	1.9 ± 0.4	1.8 ± 0.5	0.12	0.56

Compared with controls, T2DM patients demonstrated significant reductions in parasympathetic indices, including RMSSD and pNN50, along with lower HF power (p ≤ 0.001). LF power was also reduced (p ≤ 0.001). Although SDNN showed a reduction in the diabetic group, the difference was not statistically significant. The LF/HF ratio did not differ significantly between the groups. NCS findings are shown in Table 3.

**Table 3:** NCS parameters in type 2 diabetics and healthy controls Values are expressed as mean ± SD. p values were calculated using independent samples t-tests. Cohen’s d represents the effect size NCS: nerve conduction study; T2DM: type 2 diabetes mellitus; CMAP: compound muscle action potential; SNAP: sensory nerve action potential; SD: standard deviation

Nerve	NCS parameter	T2DM patients (n = 100)	Healthy controls (n = 100)	p value	Cohen’s d
Motor nerves					
Median nerve	CMAP amplitude (mV)	8.36 ± 4.73	12.56 ± 2.13	0.002	1.16
	Conduction velocity (m/s)	50.85 ± 7.84	55.48 ± 10.42	0.071	0.36
Posterior tibial nerve	CMAP amplitude (mV)	10.7 ± 1.8	13.61 ± 1.9	<0.001	1.25
	Conduction velocity (m/s)	48.82 ± 11.43	57.14 ± 11.32	<0.001	0.77
Sensory nerves					
Median nerve	SNAP amplitude (µV)	12.2 ± 3.8	18.7 ± 4.4	0.01	1.27
	Conduction velocity (m/s)	51.10 ± 9.23	53.65 ± 11.88	0.07	0.29
Sural nerve	SNAP amplitude (µV)	8.7 ± 2.5	16.73 ± 4.92	<0.001	1.85
	Conduction velocity (m/s)	41.6 ± 3.75	49.6 ± 4.68	0.01	1.50

T2DM patients exhibited significantly lower sensory and motor amplitudes compared with controls. The most pronounced reduction was observed in sural SNAP amplitude (p < 0.001). Posterior tibial and median motor CMAP amplitudes were also significantly reduced. Sensory and motor conduction velocities demonstrated modest slowing, with significant differences noted for posterior tibial and sural nerves, consistent with early length-dependent peripheral neuropathy. Correlations between HRV and NCS parameters among T2DM patients are summarized in Table 4.

**Table 4:** Correlations between HRV parameters and NCS measures in type 2 diabetes mellitus patients (n = 100) ^*^Values represent Pearson correlation coefficients (r) p < 0.05 indicates statistical significance HRV: heart rate variability; CMAP: compound muscle action potential; SNAP: sensory nerve action potential; SDNN: standard deviation of NN intervals; RMSSD: root mean square of successive differences; LF: low frequency; HF: high frequency; CV: conduction velocity; NCS: nerve conduction study

HRV parameter	Median motor CMAP (mV)	Median motor CV (m/s)	Tibial CMAP (mV)	Tibial CV (m/s)	Median SNAP (µV)	Median sensory CV (m/s)	Sural SNAP (µV)	Sural CV (m/s)
SDNN (ms)	0.45^*^	0.21	0.46^*^	0.14	0.39^*^	0.15	0.50^*^	0.25
RMSSD (ms)	0.33	0.17	0.25	0.21	0.28	0.13	0.43	0.21
pNN50 (%)	0.31	0.31	0.35	0.24	0.32	0.24	0.33	0.27
LF (ms²)	0.18	0.25	0.23	0.33	0.24	0.30	0.23	0.26
HF (ms²)	0.48^*^	0.22	0.37^*^	0.32	0.45^*^	0.29	0.61^*^	0.53^*^
LF/HF ratio	-0.23	-0.17	-0.17	-0.05	-0.21	-0.26	-0.45	-0.28

**Figure 1:**
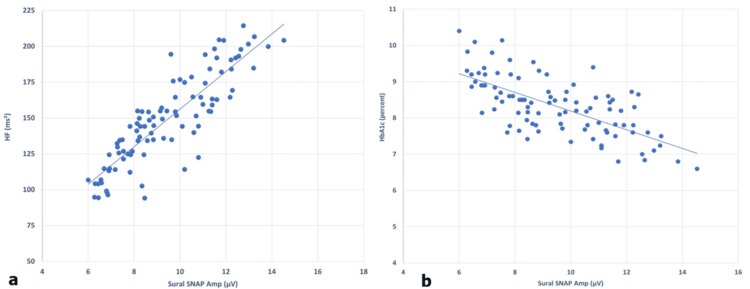
Relationship between autonomic and peripheral nerve function in type 2 diabetes mellitus. (a) Scatter plot showing a positive correlation between sural SNAP amplitude (µV) and HF power (ms²) (r = 0.61, p < 0.05). (b) Scatter plot showing a negative correlation between sural SNAP amplitude (µV) and HbA1c (%) (r = -0.66, p < 0.001) SNAP: sensory nerve action potential; HF: high frequency; HbA1c: glycated hemoglobin

Positive correlations were observed between autonomic and peripheral nerve measures. Parasympathetic HRV indices showed the strongest associations with sensory nerve amplitudes, particularly between HF power and sural SNAP amplitude (r = 0.61, p < 0.05), followed by RMSSD and sural SNAP amplitude (r = 0.50, p < 0.05). Correlations with conduction velocities were weaker and largely nonsignificant. Figure 1 illustrates the positive relationship between HF power and sural SNAP amplitude, demonstrating that better autonomic function was associated with preserved sensory nerve integrity.

Multiple linear regression analysis results are presented in Table 5.

**Table 5:** Multiple linear regression analysis of predictors of HRV parameters in type 2 diabetes mellitus patients (n = 100) Multiple linear regression analysis was performed to determine predictors of HRV parameters. β represents unstandardized regression coefficients with 95% confidence intervals CI: confidence interval; SDNN: standard deviation of NN intervals; RMSSD: root mean square of successive differences; LF: low frequency; HF: high frequency; BMI: body mass index; HRV: heart rate variability

Dependent variable	Predictor	β (95% CI)	Standardized β	p value	R²
SDNN (ms)	HbA1c (%)	-1.20 (-2.00, -0.40)	-0.35	0.006	0.31
	Diabetes duration (years)	-0.30 (-0.50, -0.10)	-0.24	0.020	
	BMI (kg/m²)	-0.15 (-0.35, 0.05)	-0.12	0.150	
	Sex (male = 1, female = 0)	0.80 (-1.00, 2.60)	0.08	0.350	
	Age (years)	-0.25 (-0.45, 0.00)	-0.23	0.050	
RMSSD (ms)	HbA1c (%)	-1.00 (-1.70, -0.30)	-0.38	0.004	0.34
	Diabetes duration (years)	-0.25 (-0.45, -0.05)	-0.26	0.030	
	BMI (kg/m²)	-0.12 (-0.30, 0.06)	-0.16	0.120	
	Sex (male = 1, female = 0)	0.60 (-1.00, 2.20)	0.07	0.350	
	Age (years)	-0.20 (-0.40, 0.00)	-0.22	0.050	
LF power (ms²)	HbA1c (%)	-15.0 (-25.0, -5.0)	-0.34	0.008	0.29
	Diabetes duration (years)	-4.0 (-7.0, -1.0)	-0.23	0.040	
	BMI (kg/m²)	-2.0 (-4.5, 0.5)	-0.14	0.140	
	Sex (male = 1, female = 0)	10.0 (-10.0, 30.0)	0.11	0.280	
	Age (years)	-3.0 (-6.0, 0.0)	-0.18	0.080	
HF power (ms²)	HbA1c (%)	-10.0 (-16.0, -4.0)	-0.36	0.005	0.32
	Diabetes duration (years)	-3.0 (-5.0, -1.0)	-0.24	0.030	
	BMI (kg/m²)	-1.5 (-3.5, 0.5)	-0.13	0.130	
	Sex (male = 1, female = 0)	5.0 (-10.0, 20.0)	0.06	0.380	
	Age (years)	-2.0 (-4.0, 0.0)	-0.19	0.070	

HbA1c and diabetes duration emerged as significant independent predictors of reduced HRV indices. For SDNN, higher HbA1c and longer disease duration were associated with lower values (model R² = 0.31, p < 0.001). Similar independent negative associations were observed for RMSSD (R² = 0.34), LF (R² = 0.29), and HF power (R² = 0.32). Age, sex, and body mass index were not significant predictors in any model.

## Discussion

CAN and DPN are common yet frequently underrecognized complications of T2DM, contributing substantially to long-term morbidity and mortality. Although clinical manifestations typically appear later in the disease course, subclinical neural dysfunction may develop during the early years of diabetes [14]. Early identification of such changes is, therefore, important for timely intervention. The present study evaluated autonomic and peripheral nerve function in patients with short-duration T2DM using HRV and NCS.

Our findings demonstrate that even within the first five years of diabetes, significant impairments are evident in both autonomic and peripheral nerve measures. T2DM patients showed reductions in parasympathetic HRV indices, particularly RMSSD and HF power, consistent with early cardiac autonomic dysfunction. In parallel, sensory and motor nerve amplitudes were reduced, with the most pronounced abnormality observed in sural SNAP amplitude, indicating early length-dependent peripheral neuropathy. These findings align with the known pattern of diabetic neuropathy, where sensory fibers and distal nerves are affected earlier than motor fibers [15].

A key observation was the positive correlation between HRV and NCS parameters, especially between HF power and sural SNAP amplitude (r = 0.62). This association suggests that autonomic and peripheral nerve impairments may progress concurrently, possibly reflecting shared pathophysiological mechanisms such as chronic hyperglycemia, oxidative stress, microvascular ischemia, and impaired neuronal repair [16]. The parallel involvement of small autonomic fibers and large somatic fibers supports the concept of a generalized neural injury in diabetes rather than isolated compartmental damage.

Diabetes-induced vasculopathy may further contribute to this parallel neural deterioration. Chronic hyperglycemia leads to endothelial dysfunction, reduced nitric oxide availability, and capillary basement membrane thickening, resulting in impaired endoneurial blood flow. Reduced perfusion of both autonomic and peripheral nerves may accelerate neural injury even in early-stage diabetes. These microvascular alterations have been implicated in early diabetic neuropathy and may explain the concurrent involvement of autonomic and somatic nerve fibers observed in the present study.

Multivariable regression analysis further demonstrated that HbA1c and diabetes duration were independent predictors of reduced HRV indices, while age, sex, and body mass index were not significant contributors. These findings highlight the central role of glycemic burden in the development of autonomic dysfunction and are consistent with earlier studies linking poor metabolic control to progressive neuropathic changes [17-19]. The observed reduction in HRV with increasing HbA1c underscores the importance of sustained glycemic control to preserve neural function. Biologically, persistent hyperglycemia leads to the accumulation of advanced glycation end products (AGEs), which form cross-links with structural proteins, lipids, and nucleic acids. These molecular alterations are particularly pronounced in collagen-rich tissues and have been linked to early nerve dysfunction. Studies utilizing skin autofluorescence as a proxy for AGE burden have shown strong associations with both autonomic and peripheral neuropathy. Additionally, HRV studies suggest that the early decline in parasympathetic tone and elevated resting heart rate seen in diabetics may reflect a shift toward sympathetic dominance rather than outright parasympathetic failure, changes that may begin even in the prediabetic stage [20].

The findings of this study may also be interpreted in the broader context of systemic diabetic complications. Diabetes-related microvascular damage affects multiple organ systems, including peripheral nerves, autonomic nerves, and renal microvasculature. Chronic hyperglycemia leads to endothelial dysfunction, oxidative stress, and capillary basement membrane thickening, resulting in reduced tissue perfusion. Similar mechanisms contribute to diabetic nephropathy, where glomerular microvascular injury occurs early in the disease course. The coexistence of neuropathy, vasculopathy, and nephropathy suggests a common microangiopathic process affecting multiple organ systems. Therefore, early autonomic and peripheral nerve dysfunction observed in this study may reflect systemic microvascular involvement in T2DM.

Previous longitudinal studies support these observations. Morimoto et al. reported progressive deterioration of nerve conduction parameters over time despite treatment, suggesting that neuropathic changes begin early and accumulate gradually [21]. Zhang et al. demonstrated that sensory nerves, particularly the sural nerve, exhibit earlier abnormalities than motor nerves [22]. Similarly, Zaidi and Gupta and Motataianu et al. documented associations between autonomic dysfunction and peripheral neuropathy using HRV and electrophysiological measures [23,24]. Our results are consistent with these reports and further reinforce the coexistence of autonomic and peripheral neural involvement in T2DM.

The combined use of HRV and NCS may therefore provide complementary information on neural integrity. HRV offers a simple, noninvasive assessment of autonomic function, while NCS provides an objective evaluation of peripheral nerve conduction. Together, these modalities may help identify early neurophysiological impairment in routine clinical settings, particularly where more complex autonomic testing is not feasible.

This study has several limitations. Its cross-sectional design precludes causal inference, and the single-center setting may limit generalizability. Inclusion was restricted to patients with a diabetes duration of less than five years; therefore, the findings may not apply to advanced neuropathy. Additionally, symptom-based assessments and clinical neuropathy scores were not included, which could have provided functional correlation with electrophysiological measures. Furthermore, potential confounding factors, such as lifestyle habits, physical activity, and associated comorbidities, were not fully controlled for and may have influenced autonomic and peripheral nerve function.

## Conclusions

This study demonstrates concurrent impairment of cardiac autonomic and peripheral nerve function in patients with early-stage T2DM. Reduced HRV was associated with abnormalities in nerve conduction measures, indicating parallel involvement of autonomic and peripheral nerves. HbA1c and diabetes duration emerged as independent predictors of autonomic dysfunction, underscoring the influence of chronic glycemic exposure on neural integrity. Combined assessment using HRV and NCS may provide complementary information for evaluating neurophysiological involvement in routine clinical practice.
